# Molecular mechanisms of metabolic dysregulation in diabetic cardiomyopathy

**DOI:** 10.3389/fcvm.2024.1375400

**Published:** 2024-03-25

**Authors:** Yue Zeng, Yilang Li, Wenyue Jiang, Ning Hou

**Affiliations:** ^1^ Key Laboratory of Molecular Target & Clinical Pharmacology, School of Pharmaceutical Sciences and the Fifth Affiliated Hospital, Guangzhou Medical University, Guangzhou, China; ^2^ Department of Pharmacy, The Sixth Affiliated Hospital of Guangzhou Medical University, Qingyuan, China

**Keywords:** diabetic cardiomyopathy, metabolism, fatty acid oxidation, glucotoxicity, heart failure, cardiac function

## Abstract

Diabetic cardiomyopathy (DCM), one of the most serious complications of diabetes mellitus, has become recognized as a cardiometabolic disease. In normoxic conditions, the majority of the ATP production (>95%) required for heart beating comes from mitochondrial oxidative phosphorylation of fatty acids (FAs) and glucose, with the remaining portion coming from a variety of sources, including fructose, lactate, ketone bodies (KB) and branched chain amino acids (BCAA). Increased FA intake and decreased utilization of glucose and lactic acid were observed in the diabetic hearts of animal models and diabetic patients. Moreover, the polyol pathway is activated, and fructose metabolism is enhanced. The use of ketones as energy sources in human diabetic hearts also increases significantly. Furthermore, elevated BCAA levels and impaired BCAA metabolism were observed in the hearts of diabetic mice and patients. The shift in energy substrate preference in diabetic hearts results in increased oxygen consumption and impaired oxidative phosphorylation, leading to diabetic cardiomyopathy. However, the precise mechanisms by which impaired myocardial metabolic alterations result in diabetes mellitus cardiac disease are not fully understood. Therefore, this review focuses on the molecular mechanisms involved in alterations of myocardial energy metabolism. It not only adds more molecular targets for the diagnosis and treatment, but also provides an experimental foundation for screening novel therapeutic agents for diabetic cardiomyopathy.

Diabetes mellitus incidence has been showing an increasing trend in recent years (1)_._ Diabetic cardiomyopathy (DCM) is characterized by abnormalities in myocardial structure and function that are unrelated to diabetic macrovascular complications such as hypertension, coronary artery disease, and atherosclerosis (2). It consists of cardiac fibrosis, cardiac hypertrophy, diastolic dysfunction, and the progression of systolic dysfunction and heart failure (3, 4). Diabetic patients have a higher rate of heart failure than non-diabetic patients (5).

Prolonged hyperglycemia promotes the accumulation of advanced glycation end products (AGEs) (6). O-GlcNAcylation, which is a post-translational modification of proteins occurring on the hydroxyl group of serine or threonine, is also increased in response to hyperglycemia (7). In diabetic hearts, the utilization and consumption of glucose decrease due to insulin deficiency or insulin resistance (8). Conversely, as fatty acids (FAs) supply and uptake increase, the heart becomes more dependent on FA for energy production (9). Furthermore, the accumulation of lipid metabolism intermediates, namely diacylglycerols and ceramides, results from unbalanced acylcarnitine synthesis and mitochondrial oxidation rate, which additionally contributes to oxidative stress, inflammation, and cardiac dysfunction (10, 11).

Some review articles, such as those written by Ding An and his colleagues, have summarized that dysregulation of glucose and lipid metabolism causes changes in cardiometabolism, which leads to mitochondrial dysfunction and impaired cardiac function (12). In addition to FA and glucose, energy can be generated using other substrates, such as fructose (13, 14), lactate (15), amino acids (16), and ketone bodies (17), in the heart. The metabolic imbalance of these fuels is also linked to the occurrence and development of DCM (Figure 1).

**Figure 1:**
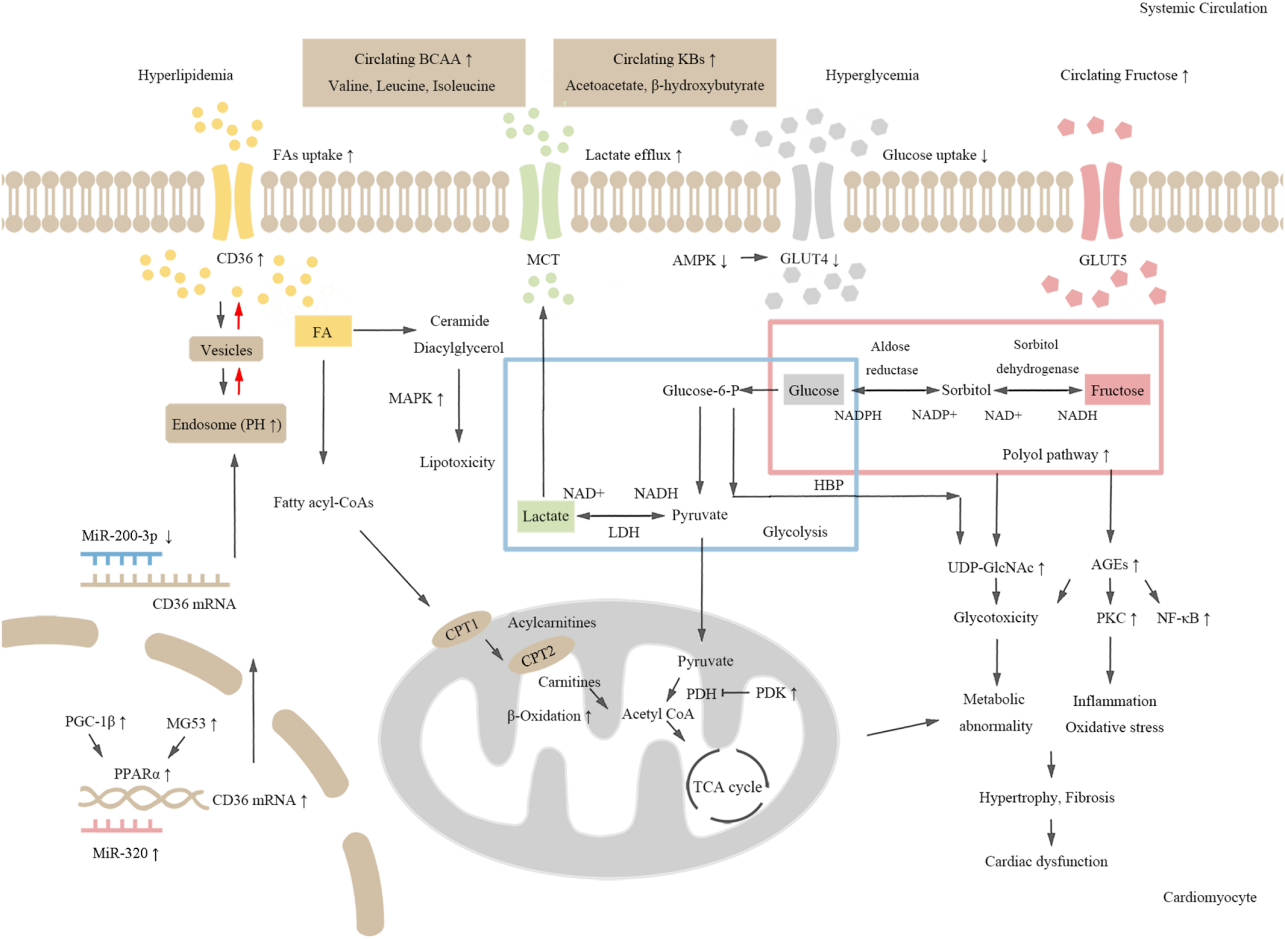
The schematic chart for molecular mechanisms of metabolic dysregulation in DCM. Hyperglycemia and dyslipidemia induce cardiometabolic disorders in diabetes. Both CD36 expression and fatty acid (FA) uptake are increased in diabetic hearts. Activated MAPK signaling pathway induces toxic lipid intermediate accumulation. Increased expression of PGC-1β and MG53, and up-regulation of PPARα and its target genes promote FA oxidation, which further leads to abnormal cardiometabolism and cardiac dysfunction. Decreased glucose and lactate uptake, activation of the polyol pathway, and enhanced fructose metabolism lead to increased production of AGEs and UDP-GlcNAc, which induce inflammation and cardiac dysfunction. Additionally, increased level of amino acids and KBs may also be risk factors for cardiometabolic disorders in diabetes.

This review focuses on changes in myocardial energy metabolism and the relevant signaling pathways connected to those modifications. Table 1 provides a summary of relevant published references on each signaling pathway. Increasing our knowledge of the molecular mechanisms that underlie metabolic disorders in DCM will help us develop effective treatment strategies.

**Table 1:** Summary of references on signaling pathways relevant to metabolic disorders in DCM.

	Associated signaling pathways and molecular targets	The influence of targets’ changes	Types of model	Cardiac pathological features	References
Fatty acid	Fatty acid transporters, CD36 (↑)	Increased fatty acid uptake	Diabetic db/db mice	Cardiac dysfunction	Li et al. (18)
	Fatty acid transporters, CD36 (↑)	Increased fatty acid uptake	STZ (40 mg/kg) induced DM rats, H9C2 cells	Cardiac dysfunction	Xu et al. (19)
	PPARγ signaling pathways (↓)	Insulin resistance	STZ-induced DM mice, NRCMs treated with glucose (HG, 40 mM)	Cardiac dysfunction and pathological remodeling	Wu et al. (20)
	PPARα signaling pathways (↑)	Increased fatty acid uptake and oxidation	Diabetic db/db mice, palmitate-treated H9C2 cells and NRCMs	Cardiac dysfunction	Yin et al. (21)
	PPARα upstream regulator, MG53(↑)	Increased lipid accumulation, compromised glucose uptake	MG53 (+/+) transgenic mice	Myocardial hypertrophy, fibrosis, and cardiac dysfunction	Liu et al. (22)
	MAPK signaling pathways (↑)	Lipotoxicity, insulin resistance	Palmitate treated human adult ventricular cardiomyocytes (AC16 cells)	Cardiomyocyte apoptosis	Oh et al. (23)
	p38 MAPK and its downstream targets, MK2 (↑)	Increased circulating levels of FFA, and cardiac triglyceride accumulation, palmitate β-oxidation	STZ induced MK2 (+/+) mice	Contractile dysfunction	Ruiz et al. (24)
Glucose	Accumulation of AGEs (↑)	Activated NF-*κ*B and PKC	STZ induced DM rats	Oxidative stress, inflammatory responses, cardiac fibrosis	Hou et al. (25)
		AGEs bind directly to MD2, leading to formation of AGEs-MD2-TLR4 complex	STZ (100 mg/kg) induced T1DM mice and db/db mice, H9C2 cells, Primary rat cardiomyocytes from SD rats, cardiac endothelial and fibroblasts from C57BL/6 mice	Derived inflammatory diabetic cardiomyopathy	Wang et al. (26)
	Hexosamine biosynthetic pathway (↑)	Lipotoxicity and glycotoxicity	T2DM mice	Cardiac fibrosis	Fricovsky et al. (27)
Lactic acid	Increased NADH/NAD^+^	Lactate efflux > lactate uptake	T1DM rats	Heart failure and myocardial remodeling	Ramasamy et al. (28)
Fructose	Activation of polyol pathway	Promotes the formation of AGEs	STZ (35 mg/kg) induced T2DM rats	Inflammation and oxidative stress	Bhattacharjee et al. (29)
BCAA	GCN2 (↑)	Lipotoxicity	STZ (50 mg/kg, five consecutive days) induced T1DM and STZ (120 mg/kg) induced T2DM mice, H9C2 cells	Hypertrophy, fibrosis	Feng et al. (30)
	Periostin/NAP1L2/SIRT3 **(↑)**	Impaired BCAA catabolism	STZ (120 mg/kg) induced DM mice, HG treated primary rat cardiomyocytes, cardiac endothelial and fibroblasts from SD rats	Hypertrophy, fibrosis	Lu et al. (31)
KBs	HMGCS2 (↑)	Balance the Acetyl-CoA/CoA ratio and increase glucose oxidation	STZ (150 mg/kg) induced T1DM rats	Improved metabolic function, cardiac dysfunction, and ventricular remodeling	Cook et al. (32)

The arrow in brackets indicate the changes of energy metabolism in diabetic cardiomyopathy. (↑), upregulation; (↓), downregulation.

## 1 Fatty acid oxidation

Disrupted lipid metabolism is an early event in diabetic heart functional abnormalities (33). McGavock et al. discovered myocardial lipid deposition in diabetic patients with normal heart function, implying that metabolic disturbances occur prior to the development of left ventricular dysfunction (33). Diabetic heart lipid metabolism is impacted due to changes in the expression of transporters involved in FA uptake (34), alterations in the PPAR signaling pathway during FA oxidation (35), accumulation of lipotoxicity, and activation of the MAPK signaling pathway (36). Lipid metabolism alterations in type 2 diabetes mellitus (T2DM) have been widely reported, and notably, lipid accumulation in type 1 diabetes mellitus (T1DM) has also been reported by relevant studies (37–39).

Heart FAs are composed of non-esterified FAs that combine with plasma albumin in the bloodstream and esterified FAs in the form of lipoprotein (40). In cardiomyocytes, 70%–90% of cytosolic FA is translocated into mitochondria for β-oxidation, which results in the production of ATP, while the remaining fraction is esterified to triglycerides (TAGs) (11). As the primary metabolic substrate, long-chain FAs (LCFAs) are esterified to fatty acyl-CoAs, which are then converted to acylcarnitines by carnitine palmitoyltransferase-1 (CPT1). Carnitine acylcarnitine translocase then transports acylcarnitines across the inner mitochondrial membrane to convert them into carnitines (41). Finally, mitochondrial FA undergoes β-Oxidation to produce acetyl CoA, which is further metabolized into the tricarboxylic acid cycle to produce ATP (41).

### 1.1 CD36

Transporters involved in cardiomyocyte FA uptake include FA translocase (FAT/CD36), FA binding protein (FABPpm), and FA transport protein (FATP) (42). FABPpm works with CD36 to facilitate FA transmembrane transport of FAs (34).

CD36 is known as a scavenger receptor that plays an important role in the uptake of long-chain FAs (34). Abnormal CD36 distribution alters myocardial energy supply, resulting to cardiac dysfunction (18). Reduced lipid uptake, attenuated lipotoxicity, and reduced cardiomyocyte apoptosis are the effects of the downregulation of CD36 in diabetic cardiomyopathy (19).

Hyperglycemia and hyperlipidemia promote CD36 translocation to the cell membrane, leading to increased FA uptake (43). Endosomes store nearly 50% of the CD36 (44). The CD36 recycling occurs in endosomes, intermediate vesicles, and at the membrane (44). The subcellular localization of CD36 is determined by the pH of the endosome, which is kept acidic by the proton pump H^+^—ATPase (V-ATPase) (45). Increased intracellular LCFA levels cause the V-ATPase subcomplex to disassemble from the intact complex, resulting in endosome alkalinization and increased relocation of CD36 to the cardiomyocyte membrane (45).

Additionally, insulin stimulation promotes CD36 translocation from endosomes to the cell membrane (43). It has been established that the vesicle-associated membrane protein (VAMP) family VAMP2, VAMP3, and VAMP4 mediate the transportation of CD36 to endosomes, intermediate vesicles, and sarcolemma (46). The only member of VAMP family of proteins, VAMP2, which is regulated by the Akt pathway, is responsible for CD36 translocation away from the sarcolemma (46). Hyperinsulinemia activates the insulin receptor substrate 1 (IRS1)/phosphatidylinositol 3-kinase (PI3K)/protein kinase B (PKB/Akt) pathways, which deactivate Akt substrate 160 (AS160) through Ser/Thr phosphorylation and subsequently reduce the downstream inhibition of Rab GTPase activating proteins (Rabs) (47). The activation of Rabs prevents the transport of CD36, which is carried out by the vesicle-associated membrane protein family (47).

FATP consists of six highly homologous proteins that are encoded by the SLC27A1-6 gene (48). LCFAs can either cross the plasma membrane directly through the FATP complex or accumulate at the plasma membrane first by binding to CD36 and then deliver FAs to FATP (49). FATP has acyl-CoA synthetase activity that stimulates the rate of cellular FA uptake by converting incoming FAs directly into their acyl-CoA thioester (48, 49).

FA uptake and lipid accumulation are increased in mice hearts that specifically overexpress FATP1 (50). Additionally, FATP6, which is primarily expressed in the heart, promotes the uptake of long-chain FAs (51). Because there is no animal model for SLC27A6, the exact role of FATP6 in the heart and other tissues is unkown (51).

### 1.2 PPAR

A key regulator of FA metabolism is the nuclear hormone receptor superfamily of ligand-activated transcription factors, known as PPAR (peroxisome proliferator activated receptor) (52). Three subtypes of PPAR exist (α, β/δ, γ) (52).

The regulation of lipid biosynthesis and insulin sensitivity is greatly influenced by the PPAR isoform PPARγ (53). The expression of PPARγ is decreased in the hearts of streptozotocin-induced diabetic rats (20). Thiazolidinediones, a type of PPARγ agonist, are used to treat type 2 diabetes mellitus (T2DM) because its effect of promoting insulin sensitization, suggesting that PPARγ activation is beneficial to ameliorate diabetic cardiomyopathy (54).

The PPARγ co-activator-1 (PGC-1) family includes PGC-1α, PGC-1β and PGC-1-related coactivator (PRC). PGC-1α and PGC-1β are involved in mitochondrial biosynthesis (53). Nuclear transcription factor (NRF), which promotes mitochondrial proliferation and regulates cellular energy metabolism, are some of the downstream factors that are stimulated by the activation of PGC-1α signal (55, 56). In the heart of T2DM db/db mice, PGC-1β expression is elevated, enhanced by the transcriptional activity of PPARα (21).

The expression of the genes responsible for FA uptake, mitochondrial FA uptake, and FA oxidation is stimulated by the activation of PPARα and PPARβ/δ (35, 52). PPARα and its target genes are upregulated in DCM, which causes increased FA uptake and decreased glucose utilization, resulting in abnormal cardiac metabolism and cardiac dysfunction (57, 58).

A novel PPARα upstream regulator called Mitsugumin 53 (MG53), also known as TRIM72, controls the expression of PPARα-encoding genes (22). MG53 is protective in cardiac ischemia/reperfusion injury, cardiomyocyte membrane injury, and cardiac fibrosis, but it simultaneously acts as an E3 ligase to promote ubiquitin dependent degradation of the insulin receptor and insulin receptor substrate, leading to insulin resistance and metabolic syndrome (59, 60).

The connection between MG53 and metabolic disorders is still debatable. According to previous studies, mice with heart-specific MG53 overexpression displayed symptoms of diabetic cardiomyopathy (22, 61). By up-regulating PPARα and its downstream targets, MG53 contributes to the pathological development of diabetic cardiomyopathy (61). Recent research, however, also revealed the opposite outcomes. According to Wang and colleagues, when compared to controls, serum MG53 levels in diabetic patients or db/db mice had decreased or remained unchanged on western blot results using a high specificity monoclonal antibody for MG53 (62, 63). The changes in MG53 may play different roles in the heart and serum according to its distribution.

### 1.3 MAPK

The state of lipids and their intermediates is dynamic. The production and accumulation of two toxic lipids, namely ceramide and diacylglycerol, may be caused by the activation of mitogen-activated protein kinase (MAPK) (36). MAPK has two subfamily members, namely p38 MAPK and c-Jun N-terminal kinase (JNK), which mediate insulin resistance and cardiac dysfunction by promoting or inhibiting the translation of target genes (36).

p38 MAPK regulates lipid and glucose metabolism, mediates insulin resistance, and contributes to diabetic cardiac dysfunction (64). Exposure to high concentrations of palmitate to mimic the lipotoxicity of diabetic hearts increased p38 MAPK phosphorylation and cardiomyocyte apoptosis (23). The activation of p38 MAPK may be associated with reduced IRS1 and IRS2 in insulin resistance. Compared with the control group, IRS1 and IRS2 protein levels and Akt phosphorylation are decreased in the hearts of diabetic mice, whereas p38 MAPK phosphorylation is increased (65).

Downregulation of p38 MAPK is beneficial for diabetic hearts. Atorvastatin improves heart function by reducing inflammation and inhibiting the activation of p38 MAPK in diabetic cardiomyopathy (66). SB203580 and SB202190, which are p38 MAPK inhibitors, reduced cell apoptosis and improved cardiac function in an animal model of STZ-induced diabetes (64).

In diabetes, the inactivation or inhibition of p38 MAPK and its downstream targets (for instance, MK2), alleviates lipid metabolism disorders and improves cardiac function (24, 67). FA oxidation and esterification is enhanced in diabetic mice, whereas the levels of free FAs are practically equal in MK2-knockout diabetic mice (MK2−/− mice) and non-diabetic mice (24).

Besides p38 MAPK, decreased JNK signaling also ameliorates diabetic cardiomyopathy (68, 69). Abnormal expression of mammalian sterile 20-like kinase 1 (MST1) is closely related to cardiac diseases (70). In db/db mice, Mst1 down-regulation protects against lipotoxic cardiac injury by inhibiting MEKK1/JNK signaling (69).

Furthermore, mammalian target of rapamycin (mTOR) is an evolutionarily conserved serine/threonine kinase involved in lipid metabolism (71). Canagliflozin (CAN), a sodium-glucose cotransporter 2 inhibitor, binds to mTOR and then inhibits mTOR phosphorylation and the expression of hypoxia inducible factor-1α (HIF-1α), reducing myocardial cellular lipotoxicity and heart injuries in diabetes (72).

## 2 Glucose oxidation

In diabetic hearts, lipid oxidation is increased while glucose oxidation is decreased (9). Members of the Sirtuins family overexpression can assist the heart's glucose oxidation efficiency (73). Glycolipid metabolism can be improved by activating AMPK signaling pathway, which promotes glucose uptake and oxidation (74).

Glucose is aerobically oxidized, producing pyruvate, which enters the mitochondria for oxidative decarboxylation. Acetyl CoA and NADH produced by β-oxidation reduce glucose oxidation through activating pyruvate dehydrogenase kinase (PDK) and inhibiting the phosphorylation of the pyruvate dehydrogenase (PDH) enzyme complex (12). This relationship between FA and glucose metabolism is called the glucose-FA cycle or Randle cycle (12, 41).

### 2.1 Sirtuins

Seven proteins constitute the Sirtuins (SIRT) are proteins that share a highly conserved NAD^+^ binding catalytic domain (SIRT1-SIRT7) (73). Sirtuin 3 (SIRT3) is a NAD^+^ dependent deacetylase, which can improve mitochondrial energy metabolism (75). According to recent research, the SIRT3 pathway promotes the shift of heart energy substrates from FA β-oxidation to glucose oxidation in DCM (75, 76). SIRT1 and SIRT6 up-regulation have also been shown to improve diabetic cardiomyopathy (77, 78).

### 2.2 AMPK

AMP-activated protein kinase (AMPK) is a myocardial glucose metabolism mediator that regulates energy metabolism exchange under cellular stress. AMPK activation increases GLUT4 expression and promotes GLUT4 redistribution to the muscular membrane, enhancing glucose uptake and improving metabolic disorders in metabolic diseases such as diabetes (74, 79). Carvacrol has previously been shown to restore GLUT4 membrane translocation mediated by PI3K/Akt signaling, lower blood glucose levels, and inhibit cardiac remodeling in both type 1 diabetes mellitus (T2DM) and type 2 diabetes mellitus (T2DM) mice (80).

Changes in the concentrations of ADP, AMP and ATP regulate AMPK. Reduced ATP production caused by hypoxia or increased energy expenditure during muscle contraction can increase cellular AMP concentrations, activating AMPK (81). AMPK activation in this scenario may favor the promotion of catabolic responses (such as FA oxidation and glycolysis) while suppressing anabolic responses (for example, FA, triglyceride, cholesterol and protein synthesis) (82, 83).

Tumor suppressor liver kinase B1 (LKB1), Ca^2+/^calmodulin-dependent protein kinase kinase β (CaMKKβ, and TGF-β activated kinase 1 (TAK1) are the three upstream kinases of AMPK (84). LKB1 directly phosphorylates AMPK THR-172 to activate its enzyme activity (85). The activation of LKB1-dependent AMPK signaling ameliorates diabetic cardiomyopathy (31, 86, 87).

An increased intracellular Ca^2+^ concentration promotes CaMKKβ-mediated AMPK activation. Adiponectin induces extracellular Ca^2+^ influx through adiponectin receptor 1 (AdipoR1), which then activates CaMKKβ and further activates the AMPK signaling pathway, playing a key role in insulin production and secretion (88).

TAK1 was discovered as the third upstream kinase AMPK activator (89). Current studies focus on its role in promoting cytoprotective autophagy through the formation of a complex with its accessory subunit TAK1 binding proteins (TAB1, TAB2, TAB3) (90). TAK1′s mechanisms of action in diabetic with metabolically disrupted hearts are unknown.

## 3 Advanced glycation end products (AGEs)

AGEs and the occurrence and progression of DCM are closely related phenomena (3, 4). AGEs accumulation is often observed in DCM disease models (91). Reducing the levels of AGEs and Receptor for AGEs (RAGE) is beneficial for structural and functional abnormalities improvement in diabetic heart (91).

AGEs are found in tissues, cells, and blood. They are the collective term for a class of stable end products formed after the free amino groups of substances such as proteins, amino acids, lipids, or nucleic acids undergo a series of reactions involving condensation, rearrangement, cleavage, and oxidative modification with the carbonyl groups of reducing sugars (6, 92).

Blood glucose levels and AGEs levels in the body are closely correlated. Persistently elevated blood sugar levels promote the glycosylation reaction between proteins and glucose, which leads to the production of AGEs in insulin deficient or insulin resistant diabetes (6).

Binding of AGEs to their receptors (RAGEs) activates multiple intracellular signaling pathways. For instance, AGEs can activate nuclear factor kappa A-β (NF-*κ*B) (25) and protein kinase C (PKC) (93, 94) signaling, which can result in the production of reactive oxygen species (ROS), inflammatory responses, and cardiac dysfunction. AGEs induced by high glucose (HG) directly bind to MD2 to form the AGEs-MD2-TLR4 complex, which starts the proinflammatory pathway, leading to inflammatory diabetic cardiomyopathy (26). Fruthermore, AGEs have the ability to alter protein structure, promote collagen cross-linking, and accelerate atherosclerosis development. The activation of AGE/RAGE signaling pathway stimulates the activaion of fibroblast and promotes fibroblast differentiation into myofibroblast, which increases extracellular matrix accumulation and accelerates pathological remodeling of diabetes heart (95).

Future treatments for the chronic diabetic complications may include blocking the AGEs-RAGE system (96). Recent studies have shown that vitamin D reduces NF-*κ*B activity, which decreases RAGE expression (97). Calcitriol has the potential to treat RAGE-mediated cardiovascular complications, because it down-regulates RAGE expression through the proteolysis of RAGE in HL-1 cardiomyocytes, mediated by disintegrin and metalloproteinase 10 (ADAM10) (97). In the DCM disease model, inhibition of protein kinase R (PKR) was found to improve diabetes-induced fibrosis by down-regulating AGEs and ERK 1/2 (98).

## 4 Hexosamine biosynthetic pathway (HBP)

The accumulation of O-linked N-acetylglucosamine (O-GlcNAc), a post-translational modification of proteins, in the heart predisposes to glucotoxicity, inducing insulin resistance (4, 99). Diabetic cardiomyopathy can be improved by HBP hyperactive suppression (100, 101).

The primary branch of the glycolysis pathway is HBP. This pathway metabolizes 2%–5% of the glucose (99). O-GlcNAcylation is a dynamic and reversible modification that primarily occurs in the cytoplasm and nucleus, in contrast to advanced glycosylation and other forms of glycosylation in the endoplasmic reticulum and Golgi apparatus (102).

When glucose enters the cell, it is phosphorylated to glucose-6-phosphate and metabolized to fructose-6-phosphate, which feeds into the glucose oxidative metabolism, glycolysis, and gluconeogenesis pathways (7). The first reaction is the rate-limiting conversion of fructose-6-phosphate to glucosamine-6-phosphate by l-glutamine-fructose-6-phosphate amidotransferase (GFAT) with conversion of glucosamine to glutamine. The second reaction involves the use of acetyl CoA as a substrate to convert glucosamine-6-phosphate to n-acetylamino-6-phosphate by glucosamine-6-phosphate acetyltransferase. After that, phosphoglucomutase converts n-acetylglucosamine-6-phosphate to n-acetylglucosamine-1-phosphate. Finally, pyrophosphorylase catalyzes the conjugation of N-acetylglucosamine to uridine nucleotides, resulting in uridine diphosphate N-acetylglucosamine UDP GlcNAc, which acts as the monosaccharide donor for o-glcnacylation. O-GlcNAc transferase (OGT) links O-GlcNAc to protein serine and threonine residues during this process. Conversely, β-n-acetylglucosaminidase (OGA) removes O-GlcNAc (102).

Under physiological conditions, transient activation of O-GlcNAc signals acts as a cellular protective mechanism (103). However, growing evidence suggests that long-term O-Glcnacylation protein elevation in diabetic animals’ hearts is associated with glucose toxicity (103, 104). Hyperglycemia induces glycogen synthase O-Glcnacylation, which reduces its activity and leads to insulin resistance. Increased HBP flux and O-Glcnacylation increased FA oxidation during glucosamine perfusion in hearts *in vitro*, implying that high O-GlcNAC levels cause both cardiac lipotoxicity and cardiac glycotoxicity (27).

Additionally, the activity of a set of proteins related to metabolic regulation, such as IRS1/2, Akt, AMPK, and GLUT4, decreases through O-GlcNAc modification. Demonstrating that O-GlcNAcylation may be a potential mechanism underlying the typical metabolic dysfunction of hearts (99).

Recent research has demonstrated that the hypoglycemic drug dapagliflozin, a sodium-dependent glucose cotransporter 2 (SGLT2) inhibitor, reduces cardiac HBP and improves diastolic dysfunction in lipodystrophic T2DM mouse models (101). In diabetic rats with significantly increased O-GlcNAcylation, thiamine may block the biosynthesis of hexosamine and prevent diabetes-induced cardiac fibrosis (100).

## 5 Fructose and lactate

Lactic acid is also a vital energy substrate for the myocardium during exercise or myocardial stress (15). Lactic acid is produced by glucose through anaerobic glycolysis (105). The discovery of monocarboxylate transporters (MCTs) laid the foundation for the study of the transmembrane transport of lactate. The MCTs consist of 14 members, and MCTs 1–4 are responsible for transporting monocarboxylates (like L-lactate and pyruvate) and ketone bodies across the plasma membrane (106). Lactate dehydrogenase (LDH) can convert lactic acid into pyruvate in normoxic conditions, providing energy for the tricarboxylic acid cycle (TCA) to produce ATP (106).

Diabetes impairs glucose and lactate metabolism in the myocardium (15). Decreased lactate uptake is associated with an increased cytosolic NADH/NAD^+^ ratio in the diabetic state (28).

Elevated fructose levels in the hearts of diabetic patients can be divided into extracellular and intracellular sources. Dietary intake is the main extracellular source of fructose, which enters the cardiomyocytes via the systemic circulation. The intracellular source is the polyol pathway, wherein glucose is reduced to sorbitol by aldose reductase and sorbitol is then oxidized to fructose by sorbitol dehydrogenase (SDH) (14).

It has been demonstrated that the proteins required for fructose transport, including GLUT5, GLUT11 and GLUT12, are expressed in the heart (13). The GLUT11 and GLUT12 have little impact on fructose transport in cardiomyocytes, while the glucose transporter GLUT5 is highly specific for fructose and has a low affinity for glucose transport (13).

The formation of fructose-derived AGEs is faster than that of AGEs derived from glucose. Persistent hyperglycemia activates polyol pathways, which cause T2DM to overproduce AGEs (107). Protocatechuic acid, a phenolic from the leaves of *Polygonum cuspidatum*, significantly suppressed AGEs levels in the serum of T2DM rats by inhibiting the activation of the polyol pathway through reducing the activities of aldose reductase and sorbitol dehydrogenase and increasing glyoxalase I activity (29).

Fructose exposure is associated with metabolic disorders, lipid accumulation, inflammation, and apoptosis (108, 109). Increased cellular fructose metabolism promotes the formation of O-Glcnacylation and AGEs, which are crucial for fructose-mediated cardiomyocyte signaling and dysfunction (13, 14).

## 6 Ketone bodies and amino acids

In the field of diabetic cardiomyopathy research, the majority of research on metabolic substrates has focused on the changes in FA and glucose metabolism. However, amino acids and ketone bodies (KB) are also used as fuel by cardiomyocytes (17, 110). Branched amino acids (BCAA) and ketone bodies produce acetyl-CoA via branched-chain alpha-ketoate dehydrogenase (BCKD) and beta-hydroxybutyrate dehydrogenase (βDH), respectively, which supplies ATP to the heart (17).

Branched chain amino acids (BCAA) are composed of valine, leucine, and isoleucine (111). In the heart, the first step of BCAA metabolism is the transamination of BCAA into the corresponding branched-chain α-ketoic acid (BCKA) by mitochondrial branched-chain aminotransferase (BCATm). The second step involves oxidative decarboxylation of BCKA by mitochondrial branched-chain alpha-ketoate dehydrogenase (BCKDH). Finally, acetyl-CoA is generated for the TCA cycle (112).

Increased BCAA levels in the blood may be a diabetes risk factor (111). Targeting the gut microbiota to reduce the abnormalities of circulating branched chain amino acids may be a key strategy to improve heart function, according to Yang and his colleagues (112).

General control nonderepressible 2 (GCN2) is an evolutionarily conserved eukaryotic initiation factor 2α (eIF2α) Kinases, which serves as an amino acid sensor (113). When amino acid levels are insufficient, GCN2 can selectively stimulate amino acid biosynthetic gene expression, maintaining amino acid homeostasis (30, 113). GCN2 deficiency in mice improves streptozotocin (STZ) or high-fat diet (HFD) induced diabetic cardiac dysfunction by reducing lipotoxicity and reducing oxidative stress (30).

BCAA also has the function of regulating signaling pathways in the heart. Continuous mTOR signaling, particularly involving leucine, impairs insulin signaling via insulin receptor substrates (IRS) (16, 114). Additionally, impaired BCAA metabolism causes toxic BCAA metabolites accumulation (110). In myocardial fibroblasts, high expression of periosteal protein upregulates nucleosome assembly protein 1-like 2 (NAP1L2) to deacetylate enzymes related to BCAA catabolism, which promotes cardiac fibrosis in diabetic cardiomyopathy (31).

Diabetes can lead to an increase in circulating ketones bodies (KBs) (17, 115). Ketogenesis is the synthesis of KBs through consuming acetyl coenzyme A (acetyl-CoA) produced by lipolysis (116). The two main KBs, acetoacetate and β-hydroxybutyrate (βHB), are essential in maintaining bioenergy homeostasis in diabetic cardiomyopathy (116). It has been established that the expression of hydroxymethylglutaryl-CoA synthase 2 (HMGCS2) is increased in T1DM hearts (32). In comparison with control rats, HMGCS2 protein expression was eight times higher in the hearts of diabetic rats (32). This suggests that the heart opposes “metabolic inflexibility” by transferring excess intramitochondrial Acetyl-CoA of FA oxidation to KBs, thereby releasing free CoA to balance the Acetyl-CoA/CoA ratio in favor of increased glucose oxidation via the pyruvate dehydrogenase complex. Enhanced ketogenesis is likely an adaptive mechanism of cardiac function in diabetic hearts (117).

Increased KBs use in T2DM may help improve cardiac energy efficiency (116). Ketone levels increase in patients with T2DM receiving SGLT-2 inhibitors, which may be associated with a reduced risk of heart failure mortality (118). The increased use of KBs in patients with heart failure or diabetes may be explained by the fact that ketone body breakdown requires less oxygen than FA oxidation to produce the same amount of ATP (119). In contrast with a control group receiving only AGE, Tao and his colleagues found that AGE plus KB treatment inhibited FA oxidation (120). However, KBs leads to DCM cardiac dysfunction and ventricular remodeling despite improvements in metabolic function (120). Therefore, there is still debate regarding how KBs utilization affects cardiac function in diabetes.

## 7 miRNA and diabetes cardiomyopathy

The pathological mechanism of diabetic cardiomyopathy is also related to the expression of non-coding RNA. MiRNA binds to the 3′ untranslated region (UTR) of messenger RNA (mRNA) molecule and regulates the expression of cardiac metabolism-related genes at the post-transcriptional level through mRNA translation inhibition or degradation (121). MiR-320 is highly expressed in diabetic cardiomyopathy mice and diabetic patients (18). It translocates to the nucleus and enhances the transcription of the FA metabolism-related gene CD36, which increases the uptake of free FA and induces myocardial lipotoxicity (18). Conversely, the expression of mir-200b-3p was decreased in DCM. Upregulation of mir-200b-3p inhibits CD36 and reduces cardiomyocyte apoptosis (19). Studies have shown that PGC-1β may be a target of miR-30c. MiR-30c reduces transcriptional activity of PPARα regulated by PGC-1β and suppresses the conversion of cardiac metabolism to FA induced by palmitate (21).

LncRNA is a specific transcript comprising over 200 nucleotides that are not translated into proteins. They bind to the miRNA through base pairing and block its regulatory function (122). Metastasis-associated lung adenocarcinoma transcript 1 (MALAT1, also named NEAT2) is a long non-coding RNA with a miR-26a-binding region in the transcriptional sequence, which is significantly upregulated in cardiomyocytes treated with palmitic acid (123). The downregulation of MALAT-1 inhibits the TLR4/NF-*κ*B signaling pathway by regulating HMGB1 expression, which may be the potential mechanism to relieve the inflammatory response and decrease myocardial lipotoxic injury (123).

## 8 Limitation and prospect

At present, the research into metabolic disorders in diabetic cardiomyopathy is comprehensive and extensive. The changes in some signaling pathways and molecular targets have been relatively clear, but there are still some limitations that cannot be ignored.

To begin, the signal pathways underlying metabolic disorders in DCM crosstalk with one another, and the relevant molecular mechanisms are complex. For example, CD36 inhibits AMPK activation by forming a molecular complex with Lyn and LKB1 and then participates in the energy regulation process (124). PGC-1α not only regulates FA β-oxidation and excess myocardial lipid accumulation, but it also promotes the AMPK regulated expression of several key players in mitochondrial and glucose metabolism (55, 56).

Second, the multiple pathophysiological mechanisms involved in the development of DCM remain controversial. There is no agreement on whether changes in some signaling pathways and molecular targets (such as HBP and MG53) are beneficial or harmful in the process of disease progression has not yet reached a consensus. More experimental research and theoretical support are required.

In recent years, there has been an increase in the number of studies focusing on mitochondrial dysfunction. The balance between mitochondrial biogenesis and mitophagy is critical for maintaining cellular metabolism in the diabetic heart (125, 126). A comprehensive study of the mitochondrial quality control (MQC) system, which includes mitochondrial fission, fusion, and mitophagy, may shed new light on cardiometabolic disorders in DCM.

Besides, previous research by our group has shown that overexpression of the Hippo pathway effector YAP promotes cardiac remodeling and cardiac fibrosis, leading to cardiac dysfunction (127, 128). Additionally, it may also participate in the glycosylation process and affect diabetic cardiometabolism. However, more investigation into the relationship between these molecular and metabolic disorders in DCM is required. A greater understanding of diabetes and the complications associated with cardiovascular disease will result from an in-depth investigation of these signaling and pathological pathways in myocardial metabolic disorders.
